# Supplementary material for: Morphometric geometric differences between right and left human tali: A cadaveric study of fluctuating asymmetry via systematic measurement and three-dimensional scanning
Source: PLoS One. 2020 Apr 22;15(4):e0232012. doi: 10.1371/journal.pone.0232012 (PMC7176106; doi:10.1371/journal.pone.0232012)
Supplement: Attachment — Submitted filename: Letter of Revision - Plos One.docx [file pone.0232012.s002.docx]

**Letter of Revision**

First of all, thank you for your comments about our paper. We have revised our paper in accordance with the comments as our responses in blue letters as below.

1. In the ethics statement in the manuscript and in the online submission form, please provide additional information about the human tissues used in this study. Specifically, please ensure that you have discussed whether next-of-kin provided informed written consent for the use of the tissues. If patients provided informed written consent prior to death to have their bodies used in medical research, please include this information.

Response: We have revised this statement in line numbers 124-128 in accordance with your suggestion.

2. Thank you for including your ethics statement: 'This study was approved by the ethical committee of the institution's author.'

a) Please amend your current ethics statement to include the full name of the ethics committee/institutional review board(s) that approved your specific study.

Response: We have revised this statement in line numbers 125, 126 in accordance with your suggestion.

b) Once you have amended this/these statement(s) in the Methods section of the manuscript, please add the same text to the “Ethics Statement” field of the submission form (via “Edit Submission”).

Response: We have revised this statement in accordance with your suggestion in the submission form.

For additional information about PLOS ONE ethical requirements for human subjects research, please refer to http://journals.plos.org/plosone/s/submission-guidelines#loc-human-subjects-research.

3. Thank you for including the following funding information within the acknowledgements section of your manuscript; "The author would like to propose the special thanks to Thammasat University that provide the research grant for this research project (grant number: ทป2/43/ 2561, year 2018)"

We note that you have provided funding information that is not currently declared in your Funding Statement. However, funding information should not appear in the Acknowledgments section or other areas of your manuscript. We will only publish funding information present in the Funding Statement section of the online submission form.

Please remove any funding-related text from the manuscript and let us know how you would like to update your Funding Statement. Currently, your Funding Statement reads as follows:

"Dr. Angthong reports grants from Thammasat University, during the conduct of the study; other from Amgen, personal fees from Phoenix Surgical Equipment (Thailand) Co., Ltd, outside the submitted work; In addition, Dr. Angthong has a patent Manufacture protocol for the artificial total talus issued"

Response: We have revised this statement at the acknowledgement in line numbers 271, 272 in accordance with your suggestion. We have informed you about our updated Funding Statement via a revised cover letter.

4. a. Thank you for including your funding statement; "Dr. Angthong reports grants from Thammasat University, during the conduct of the study; other from Amgen, personal fees from Phoenix Surgical Equipment (Thailand) Co., Ltd, outside the submitted work; In addition, Dr. Angthong has a patent Manufacture protocol for the artificial total talus issued"

We note that you received funding from a commercial source: AMgen and Phoenix Surgical Equipment (Thailand) Co., Ltd

Please provide an amended Competing Interests Statement that explicitly states this commercial funder, along with any other relevant declarations relating to employment, consultancy, patents, products in development, marketed products, etc.

Within this Competing Interests Statement, please confirm that this does not alter your adherence to all PLOS ONE policies on sharing data and materials by including the following statement: "This does not alter our adherence to PLOS ONE policies on sharing data and materials.” (as detailed online in our guide for authors http://journals.plos.org/plosone/s/competing-interests). If there are restrictions on sharing of data and/or materials, please state these. Please note that we cannot proceed with consideration of your article until this information has been declared.

Response: We have amended our COI document in accordance with your suggestion. We have informed you about this amendment via a revised cover letter.

b.

We note that you have a patent relating to material pertinent to this article. Please provide an amended statement of Competing Interests to declare this patent (with details including name and number), along with any other relevant declarations relating to employment, consultancy, patents, products in development or modified products etc. Please confirm that this does not alter your adherence to all PLOS ONE policies on sharing data and materials, as detailed online in our guide for authors http://journals.plos.org/plosone/s/competing-interests by including the following statement: "This does not alter our adherence to PLOS ONE policies on sharing data and materials.” If there are restrictions on sharing of data and/or materials, please state these. Please note that we cannot proceed with consideration of your article until this information has been declared.

This information should be included in your cover letter; we will change the online submission form on your behalf.

Please include your amended Competing Interests Statement within your cover letter. We will change the online submission form on your behalf.

Response: We have amended our COI document in accordance with your suggestion. We have informed you about this amendment via a revised cover letter.

Please know it is PLOS ONE policy for corresponding authors to declare, on behalf of all authors, all potential competing interests for the purposes of transparency. PLOS defines a competing interest as anything that interferes with, or could reasonably be perceived as interfering with, the full and objective presentation, peer review, editorial decision-making, or publication of research or non-research articles submitted to one of the journals. Competing interests can be financial or non-financial, professional, or personal. Competing interests can arise in relationship to an organization or another person. Please follow this link to our website for more details on competing interests: http://journals.plos.org/plosone/s/competing-interests

5. Please amend your supporting information COI files to 'other' as these need not be included within your published manuscript

Response: Supporting information files has been amended as ‘other’ in accordance to your comment.

[Note: HTML markup is below. Please do not edit.]

Reviewers' comments:

Review Comments to the Author

Reviewer #1: In this study, a total of 24 tali were measured with a vernier caliper and three-dimensional laser scanner. The significant differences in the morphometric profile were found between the right and left matched pairs of tali. However, there are some points that need to be addressed.

1. Is it possible that the differences in the morphometric profile between the right and left matched pairs of tali due to the samples of elderly cadavers？

Response: We have revised this statement in line numbers 223-232 in accordance with your suggestion.

2. Please use a unified decimal place in the tables.

Response: We have revised the table 1 to table 4 in accordance with your suggestion and unified decimal has been placed in all the tables.

Reviewer #2: In this study, the authors demonstrated the morphometric geometric variations between the right and left tali in matched pairs of human cadaveric specimens. There are a few concerns on the methodology and interpretation of the findings.

1. In addition to age, gender and height, we understand that the bone morphometry especially for calcaneum and talus, also depends on the occupation and weight. Did the authors consider these factors as well?

Response: We have revised this statement in line numbers 257-262 in accordance with your suggestion.

2. From line 134 to line 136, the authors mentioned that 'Differences in categorical data were analyzed using the chi-square … Pearson's correlation coefficient was used to express the correlation between the parametric parameters'. In the Result section, I couldn't find the findings using correlation analyses or analyses of categorical data. Is this part of analysis missing in the Result section?

Response: There was no analysis about categorical data in this study. The statement as ‘Differences in categorical data were analyzed using the chi-square test or Fisher’s exact test’ was removed from line numbers 138, 139.

3. From line 201 to line 207, the authors cited several articles using different methods to measure the talus morphometry. However, they didn't describe whether their findings are similar/different from the others. What is the purpose of citing these articles?

Response: We have described the statement in line numbers 206-214 in accordance with your suggestion.

4. It is not clear what 'stressful conditions' the authors are referring to in line 213.

Response: We have revised this statement in line numbers 216-223 in accordance with your suggestion.

5. In Table 1, five out of seven parameters of tali showed that right tali were larger than left tali. It seems that there is right dominance of talus morphometry, like handedness. The authors did not discuss the potential relationship between limb dominance and talus morphometry.

Response: We have revised this statement in line numbers 232-237 in accordance with your suggestion.

6. The clinical significance is not clear. Ankle arthroplasty implant definitely requires the measurements on the articulations of the bones surrounding the pathologic/injured talus. It is not clear how the findings in this study contribute to the improvement of current technology of making the implants.

Response: We have revised this statement in line numbers 241-244 and 245-252 in accordance with your suggestion.
